# Can we expect an increased suicide rate due to Covid-19?

**DOI:** 10.1017/ipm.2020.46

**Published:** 2020-05-21

**Authors:** Patrick Devitt

**Affiliations:** Department of Psychiatry, University of Limerick, Limerick, Ireland

**Keywords:** War, disaster, economic recession, suicide rate, Covid-19

## Abstract

Human disasters come in all shapes and sizes including wars, terrorist violence, natural events, economic recessions and depressions as well as infection. As a species more fragile than we often allow, humans would be expected to adversely react to these types of disasters in terms of mental ill health and possibly suicidal behaviour leading to increased demands on the Mental Health services. This narrative historical paper examines relevant studies into how previous disasters affected mental health and suicidal behaviour. The characteristics of what is known of the current Covid-19 disease are analysed and compared to other types of disasters with a view to gaining some insight into what we might expect. Of all the types of disasters, economic recession appears most toxic. Mitigating the worst effects of recession appears to be protective. Particularly vulnerable groups are identified in whom we might expect an increase in suicidal behaviour.

Human disasters come in all shapes and sizes including wars, terrorist violence, natural events, economic recessions and depressions as well as infection. Ireland has experienced its own share of these types of disasters including in the last century our war of independence, the civil war, ‘the troubles’ in Northern Ireland and the 2008 to 2012 economic meltdown. Our temperate climate and geographical location have largely saved us from natural disasters such as earthquakes, hurricanes and widespread flooding. As a species more fragile than we often allow, humans would be expected to adversely react to these types of disasters in terms of mental ill health and possibly suicidal behaviour leading to increased demands on the mental health services.

This paper will examine relevant studies into how previous disasters affected mental health and suicidal behaviour. The characteristics of what is known of the current Covid-19 disease will be analysed and compared to other types of disasters with a view to gaining some insight into what we might expect.

## What causes suicide?

What provokes an individual to engage in an act of suicide on any given date and time is not straightforward. It is likely that such a decision and act comprises ‘a perfect storm’ which includes the presence of mental illness, perhaps a genetic pre-disposition, family history, certain personality traits (such as impulsivity), the availability of means, the abuse of alcohol or other substances and a recent insoluble predicament (Pridmore, 2010).

In population terms, certain associations are well known including mental illness, substance abuse, availability of the means of suicide as well as social and cultural factors. Emile Durkheim, around the end of the 19th century, offered the first comprehensive theory of suicide (Durkheim, 1897). He linked the apparent rise in suicide rates at the time to modernity and the associated weakening of family and community bonds. It was Durkheim’s view that linking suicide almost exclusively with mental illness was hopelessly inadequate and completely ignored potent social forces.

Durkheim described four types of suicide, two on each of two axes. Too much social integration resulted in the altruistic suicide and not enough, the egotistic suicide. In terms of regulation of society, too much control resulted in fatalistic suicide and insufficient control led to anomic suicide due to alienation from society. It was Durkheim’s view that suicide was reduced during wars because of the greater social integration. It is now generally accepted that the presence of mental illness is a major factor but not the exclusive factor in the incidence of suicide.

## War and violence

### World War II

Durkheim’s view that war reduces suicide through greater social and political integration was examined in the United States between 1933 and 1976. It was concluded, ‘An examination of trends and suicide rates among white US adults does not show, however, that war directly decreases the suicide rate…’ (Marshall, 1981).

Though all-age male and female suicide rates decreased in Scotland during World War II, taking account of the prior background declining trend (in 1931, 20.7/100,000; in 1952, 11.3/100,000), it was concluded that the rate of suicide was higher than it would otherwise have been. The figures concealed large increases in the suicide rate in young men and an increase of suicide by firearms (Henderson *et al.* 2006).

### ‘Troubles’ in Northern Ireland

‘The troubles’ in Northern Ireland up to the signing of the Belfast or Good Friday Agreement in 1998 were associated with a suicide rate half of that after that date, from about 150 deaths per year (approx. 10/100,000 population) in the mid-1990s to more than 300 deaths (approx. 20/100,000) by the year 2010. This was thought to be related to the ‘growing of the social economic and political legacy of the troubles and in particular of the transgenerational effect of conflict-related trauma on the mental health of the population…’ (O’Connor & O’Neill, 2015). It was argued that high suicide rates in some areas might have been because of the loss of community connectedness and sense of purpose that was evident during the troubles in addition to the co-existence of many traditional risk factors as well as conflict exposure (O’Connor & O’Neill, 2015).

The turning inwards of aggression in depression can lead to suicide, and when turned outwards, the suicide rate decreases. This was suggested as a possible cause of the relative lack of psychiatric morbidity and suicide during the troubles (Curran, 1988).

### The 11th September 2001 terrorist attacks in the USA

Studies of the effects of these attacks have reached quite variable conclusions. The decrease in British suicide rates noted the month after the attacks was thought to be a demonstration of Durkheimian social principles (Salib, 2003). However, in the Netherlands in the months following 9/11, fatal and non-fatal suicidal behaviour rates rose (De Lange & Neeleman, 2004). In New York, a temporary drop was noted along with a temporary rise in other locations and an overall reasonably constant suicide rate in the United States (Claasen *et al.* 2010). In Germany, no evidence of an increase in suicide rate was found. ‘There was no evidence of Durkheim’s theory attributable to the 9/11 attacks was found in the sample…’ (Medenwald, 2016).

### The 7th July 2005 terrorist attacks in London

In the days following the 7th July 2005 attack and also on the days following 21st July 2005 second wave, brief but significant reductions in suicides were seen that had not been seen during the same period in the previous 4 years. No evidence was found of any longer-term effect on suicide (Salib & Cortina-Borja, 2009).

### Summary

As shown in Table 1, the evidence for the effect of war and violence on suicide rate is quite mixed. The increased rate in young men seen in Scotland during World War II appears most persuasive.

**Table 1.** War and violence summary

Event	Study	Suicide rate	Comment
War (in general)	Durkheim (1897)	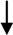	Accuracy of study unclear
World War I	Wasserman (1992)	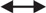	Accuracy of study unclear
World War II	Marshall (1981)	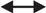	US study
	Henderson (2006)	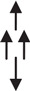	Interrupted trend of suicide decline Young men
‘Troubles’ Northern Ireland	O’Connor & O’Neill (2015)		Doubling of rate after ‘Troubles’
‘9/11’ Terrorist Attack NYC	Salib (2003)	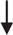	UK Only in month after attack
	DeLange & Neeleman (2004)	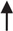	Netherlands In months following
	Claassen (2010)	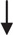 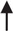	Temporary drop in NYC Temporary rise other US locations
	Medenwald (2016)	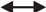	Germany
7 July 2005 21 July 2005 London Terrorist Attacks	Salib & Cortina-Borja (2009)	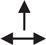	In days after Longer term

## Natural disasters

A comparison of the prevalence and characteristics of suicide following the January 2011 Queensland Australia floods to the 11 years prior for the same period found no increase in suicide rates during the 6 months after the floods. However, a delayed effect was not discounted and ongoing surveillance was recommended. In a personal communication to the author, it appeared there was no significant increase in suicide following the earthquake in New Zealand on 22 February 2011 (De Leo *et al.* 2013). Significant increases in the prevalence of suicidal ideation and suicide plans 6 months after Hurricane Katrina (August 2005) compared to 2 years later were noted (Kessler *et al.* 2008). In Puerto Rico, from an average of 19 suicides per month (7/100,000) in the 8 months before Hurricane Maria in 2017 the rate rose to 25 per month (9/100,000) in the immediate 3 months afterwards (Preliminary Statistics of Cases of Suicide in Puerto Rico, 2017).

## Epidemics

### Severe acute respiratory syndrome

The severe acute respiratory syndrome (SARS; Coronavirus-1) in 2003 did not give rise to a pandemic but was responsible for a number of epidemics notably in Hong Kong. A study of the longer-term morbidity in a SARS survivor cohort concluded that the outbreak should be regarded as a mental health catastrophe (Mak *et al.* 2009). Other studies showed a spike in the suicide rate especially among persons age 65 and over in Hong Kong in 2003, a 31.7% increase from 2002 (Yip *et al.* 2005; Cheung *et al.* 2008).

In a further study, the suicide motives among SARS-related suicide deaths were found to be associated with stress over a fear of being a burden to their families during the negative impact of the epidemic. Social engagement, mental stress and anxiety at the time of the SARS epidemic among a certain group of older adults resulted in exceptionally high rate of suicide deaths (Yip *et al.* 2010).

### ‘Spanish flu’

Little direct data on any psychiatric surge is available from the 1918 pandemic. However, by computing monthly suicide and mortality rates and matching with exogenous social and political events, an estimate of the impact of these events on suicide behaviour in the United States between 1910 and 1920 concluded: World War I did not influence suicides; the great influenza epidemic caused it to increase; and the continuing decline in alcohol consumption between 1910 and 1920 depressed national suicide rate (Wasserman, 1992).

Analysis of the psychiatric aspects of a future avian flu pandemic concluded that such a pandemic would result in an increase in required psychiatric services with a huge increase in hospital admission, increased mortality as well as increase in delirium symptoms due to high fever. No comment was made on the effect on the suicide rate (Rissmiller, 2007).

## Economic recessions/depressions

### The Great Depression (1929–1933)

This economic depression began on Tuesday, 29 October 1929, with the famous stock market crash and lasted until 1933 spreading from the United States to most other developed economies. Between 1928 and 1932, a 22.8% increase in suicides was reported in the United States, the largest increase in any 4-year period from 1938 to 2007 (Luo *et al.* 2011).

### Asian economic crisis, 1998/1999

In 1999, during this economic crisis, suicide rates soared – 39% in Japan, 44% in Hong Kong and 45% in South Korea. Taiwan and Singapore which were not as severely affected showed no link between suicide rates and economic difficulties (Chang *et al.* 2009).

### Impact of the economic recession and subsequent austerity on suicide and self-harm in Ireland, 2008 to 2012

One study found that by the end of 2012 the suicide rate for males was 57% higher than if the pre-recession trend continued but almost unchanged for females (Corcoran *et al.* 2015). Male and female self-harm rates were 31% higher. It was concluded that ‘five years of economic recession and austerity in Ireland have had a significant negative impact on rates of suicide in men and on self-harm in both sexes…’ (Corcoran *et al.* 2015).

### Links between unemployment, suicide and social supports

A study linking unemployment and mortality in 26 European Union countries found that Finland and Sweden were outliers in that increases in suicide did not parallel unemployment. The authors speculated that active supportive labour-market programmes were partly responsible (Stuckler *et al.* 2009).

## Aspects of Covid-19 relevant to the possible development of increase in suicide

Covid-19 has hit the world including Ireland in a shockingly abrupt manner. An exponential increase in cases has necessitated Draconian government measures to slow the spread of the virus and mitigate its effects with respect to the availability of healthcare services. A raft of ‘stay-at-home’ and similar demands has plunged the economy into recession.

How are people reacting to this rapid and profound change in their lives? Some were in denial until virtual ‘lockdown’ was imposed. Others are wracked with anxiety, especially the elderly with respect to contracting the virus and ending up in an ICU bed. Many have lost jobs and perhaps livelihoods. Many are working from home along with spouses and children.

Increase in domestic violence has been reported (McGee, 2020). Anecdotal reports suggest that people are drinking more and are advised to guard against addiction (*Irish Times*, 2020). It is uncertain how long these restrictions will last. As yet, there is no measurable or observable effect on mental health services as it is likely that individuals suffering from anxiety will steer clear of general practitioners who are overwhelmed with dealing with the effects of the virus. People are also reluctant to attend emergency departments.

The impact on healthcare workers will be particularly profound. Working long hours, risking contracting the virus themselves and witnessing the overwhelming of the ICU services will likely extract a psychological toll. Further, doctors and nurses will be exposed to the risk of ‘moral injury’ with respect to decisions to deprive some individuals of care they otherwise would have provided.

Is the current pandemic akin to a war, natural disaster or other epidemics? Is the economic recession we are experiencing and will experience in the future akin to the 2008–2012 recession? As overused is the war metaphor, similarities do exist in terms of the long and uncertain timeline, the frequent strategy and mortality bulletins, economic impact and emergency, previously unthinkable, legislation as well as sending our soldiers (healthcare workers) to the front. Natural disasters are usually acute in onset and duration but health and disease after-effects resemble aspects of this pandemic. As appears obvious, this pandemic is probably closest to previous epidemics and second only in modern times to the 1918 ‘Spanish flu’ and in medieval times to plague.

Applying Durkheim’s social integration explanation for the apparent reduction in suicides during war, it does appear that there is a widespread sense of solidarity, in particular with respect to social distancing and a sense of tolerating these restrictions for the collective good. On that basis, we might expect a decrease in suicides while the emergency persists. However, the Hong Kong SARS experience alerts us to the possibility of an increase in the suicide rate of the elderly (already recognised as a vulnerable group in terms of suicide) who are disproportionately affected with respect to ‘cocooning’, severity of illness and mortality. The elderly cannot be unaffected by certain musings in the United States and the United Kingdom regarding their expendability so as not to injure the economy.

From an economic perspective, the financial protections provided by the government will mitigate the expected ill effects of sudden poverty. However, these measures are temporary and it is likely that recession will continue for some time though, as most commentators believe, not as long as the 2008–2012 recession. In addition, it may be a different type of recession in that it has been externally imposed and ‘not our own fault’ as had been the common wisdom during the previous recession – ‘we all partied’. Is it likely that people and social groups are more able to tolerate hardships that are externally imposed such as some wars and natural disasters? Perhaps.

Similar to the Northern Ireland and Hong Kong studies discussed above, when this crisis has passed, it may well be that we will see an increase with respect to the development of mental illnesses such as anxiety and Post Traumatic Stress Disorder, with some associated increase in suicidal behaviour.

## Conclusion

Humans are always prone to disasters which, in general, have an adverse effect on their mental health. Of all the types of disasters – war, violence, natural disasters, epidemics/pandemics and economic recession – it appears that the most toxic is that of economic recession. Mitigating the worst effects of a recession appears to be protective. The current financial protection measures in this regard should continue.

When the dust has settled, we may not see a major increase in pre-existing suicide-rate patterns. However, previous experience suggests that we may well see an increase in psychiatric presentations and short-term spikes in suicidal behaviour. Particularly vulnerable groups include healthcare workers, the elderly and those who suffer crushing economic adversity. Consideration should be given to prioritising these groups for ongoing mental health surveillance and treatment if necessary.
